# Supplementary material for: Simple synthesis of massively parallel RNA microarrays via enzymatic conversion from DNA microarrays
Source: Nat Commun. 2022 Jun 30;13:3772. doi: 10.1038/s41467-022-31370-9 (PMC9246885; doi:10.1038/s41467-022-31370-9)
Supplement: Supplementary file 1 — Supplementary Information [file 41467_2022_31370_MOESM1_ESM.docx]

Supplementary Material

for

**Simple synthesis of massively parallel RNA microarrays via enzymatic conversion from DNA microarrays**

Erika Schaudy^1^, Kathrin Hölz^1^, Jory Lietard^1^ and Mark M. Somoza^1,2,3✉^

^1^Institute of Inorganic Chemistry, University of Vienna, Josef-Holaubek-Platz 2, 1090 Vienna, Austria. ^2^ Chair of Food Chemistry and Molecular Sensory Science, Technical University of Munich, Lise-Meitner-Straße 34, 85354 Freising, Germany. ^3^ Leibniz-Institute for Food Systems Biology at the Technical University of Munich, Lise-Meitner-Straße 34, 85354 Freising, Germany.

✉email: mark.somoza@univie.ac.at

**Supplementary Figures**


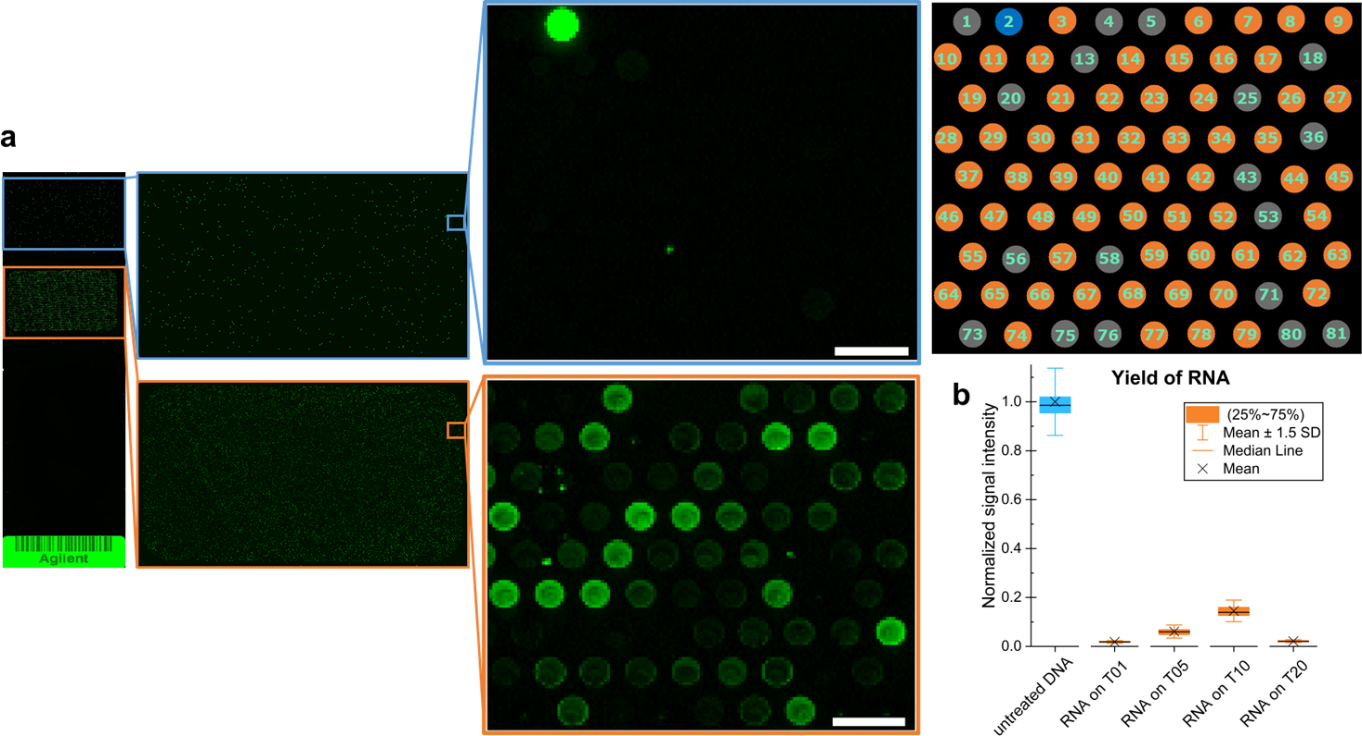


**Supplementary Figure 1**. Enzymatic conversion of a 4×44K Agilent SurePrint custom DNA microarray to an RNA array. a) Close-up of an untreated (blue frame) array and an array after conversion to RNA (orange frame) following hybridization with the probe t-Cy3 for visualization (scale bar: 100 µm), including a scheme indicating location of spots with sequences allowing for conversion to RNA (orange), hybridization to DNA (blue) and controls with unrelated sequence or background features (grey). A table with the sequences for each individual spot shown according to the numbering in the scheme is provided in the Supplementary Data File. b) Yield of the conversion process based on fluorescent signal intensities upon hybridization to RNA (orange) normalized to the signal for detection of DNA (blue) in an untreated array (60 replicates).

| 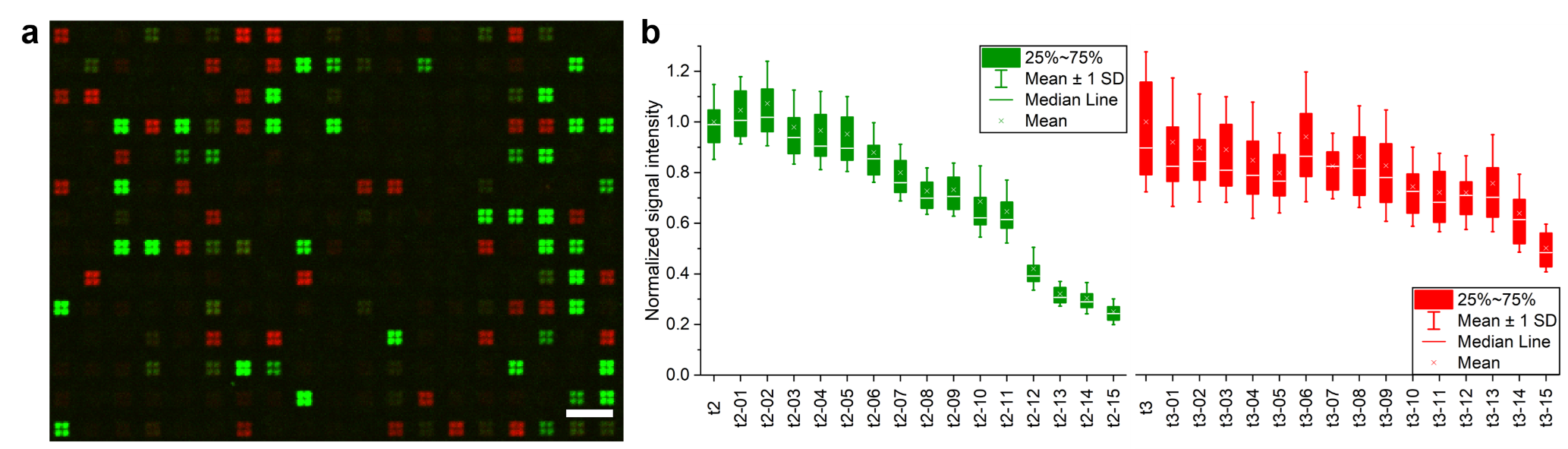 |
| --- |
| **Supplementary Figure 2**. Investigation on the transcription of truncated templates. a) Detail of a scan after simultaneous hybridization with t2-Cy3 and t3-Cy5 to an array with truncated template sequences after conversion to an RNA microarray (scale bar corresponds to 100 µm). The sequences of t2-Cy3 and t3-Cy5 oligonucleotides are shown in **Supplementary Table 3**. b) The corresponding fluorescence data for truncations of 1 to 15 nucleotides normalized to the full-length sequence (54 replicates). |

| 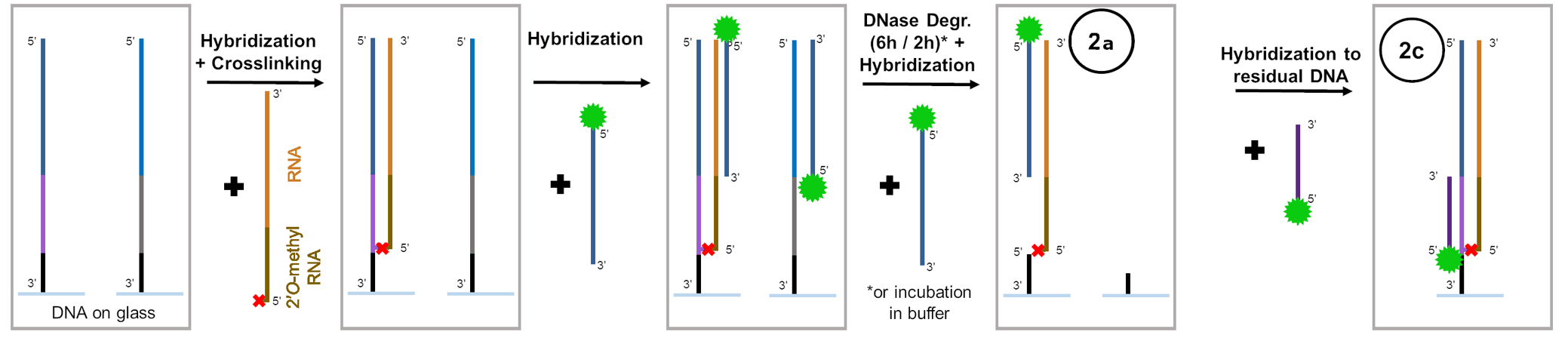 |
| --- |
| **Supplementary Figure 3**. Detailed scheme of the experimental steps performed to test DNase-mediated degradation in a system mimicking successful primer extension by hybridization and crosslinking of a 5′-psoralen-modified oligonucleotide consisting of 2′-*O*-methyl RNA (corresponding to the primer) and RNA (corresponding to the product of conversion). The experiments shown here yield the results shown in the plots in Figures 2a and 2c. |

| 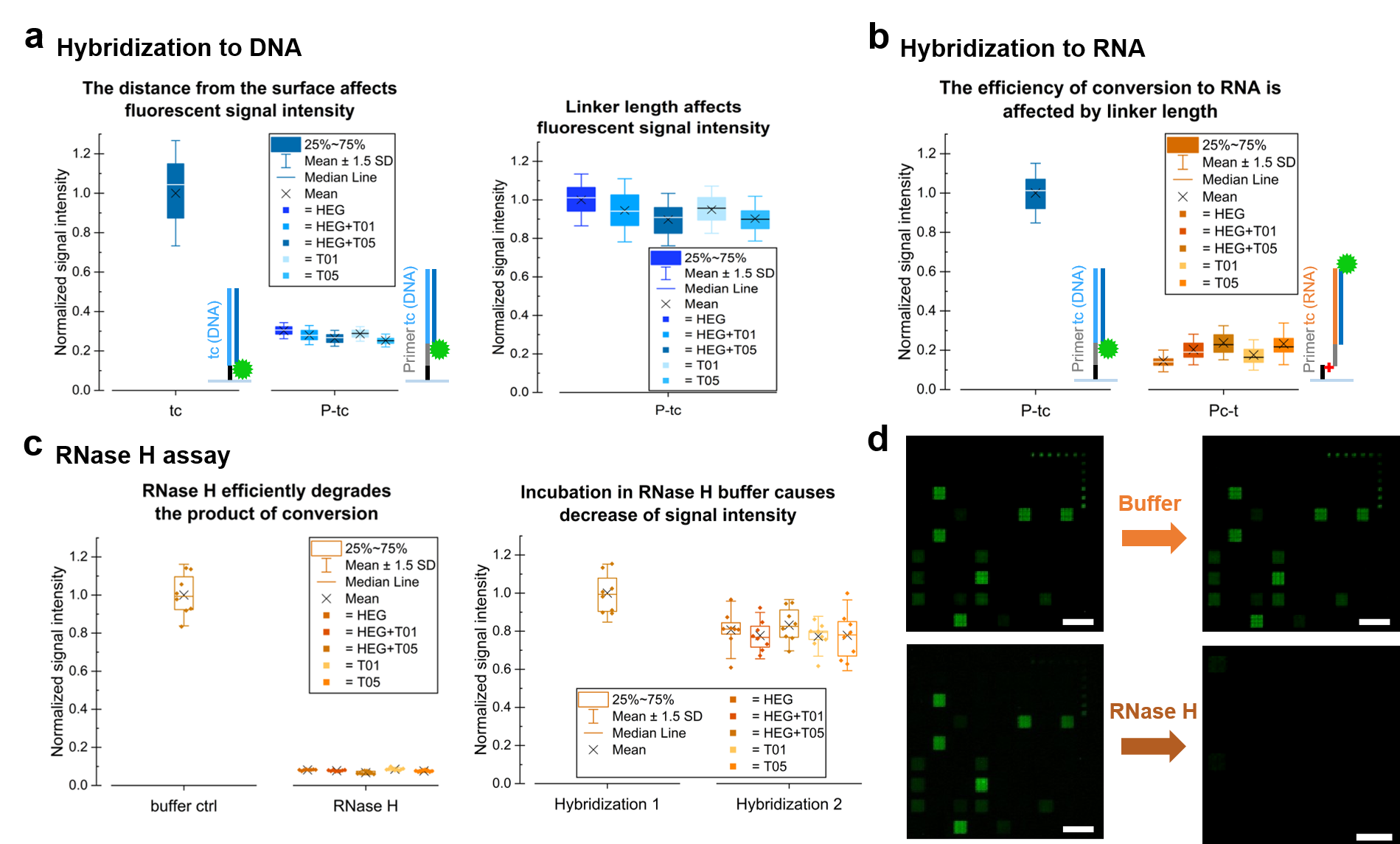 |
| --- |
| **Supplementary Figure 4**. Hybridization-based detection and degradation of RNA. a) The fluorescent signal intensities for hybridization with t-Cy3 to complementary DNA (blue) of an untreated array are affected by the distance of the target sequence “tc” to the glass surface, as can be seen by comparing hybridization efficiency to “tc” directly 5′ to the linker vs introducing a 15 nt (primer) sequence between linker and target (“P-tc”, left plot), and comparing signal intensities for hybridization to the “P-tc” construct on linkers of various lengths (n = 63 for linker lengths “HEG” and “HEG+T”, n = 62 for other linkers). b) Hybridization-based detection of RNA (orange) as the product of conversion on different linkers normalized to the signal obtained for hybridization to DNA on an untreated array (blue) (n = 63 for linker lengths “HEG” and “HEG+T”, n = 62 for other linkers). Note that the 5′-Cy3 on the complementary strand is closer to the surface when hybridizing to control DNA than when hybridizing to polymerized RNA. Dye fluorescence intensity is known to be distance-dependent. c) Comparison of hybridization to RNA upon RNase H treatment vs incubation in buffer for different linker lengths (left plot) to confirm the identity of the product of transcription. Incubation in buffer is also accompanied with loss of fluorescence (compare “Hybridization 1” vs “Hybridization 2”) indicating H_2_O-mediated loss of surface-bound oligonucleotides (right plot) (n = 9 for linker T1, n = 8 for other linkers). d) Excerpt of scans of an RNA microarray treated either with buffer (top) or with RNase H (bottom) (scale bar: 100 µm). (t(c) … template (complement), P(c) … primer (complement)). |

| 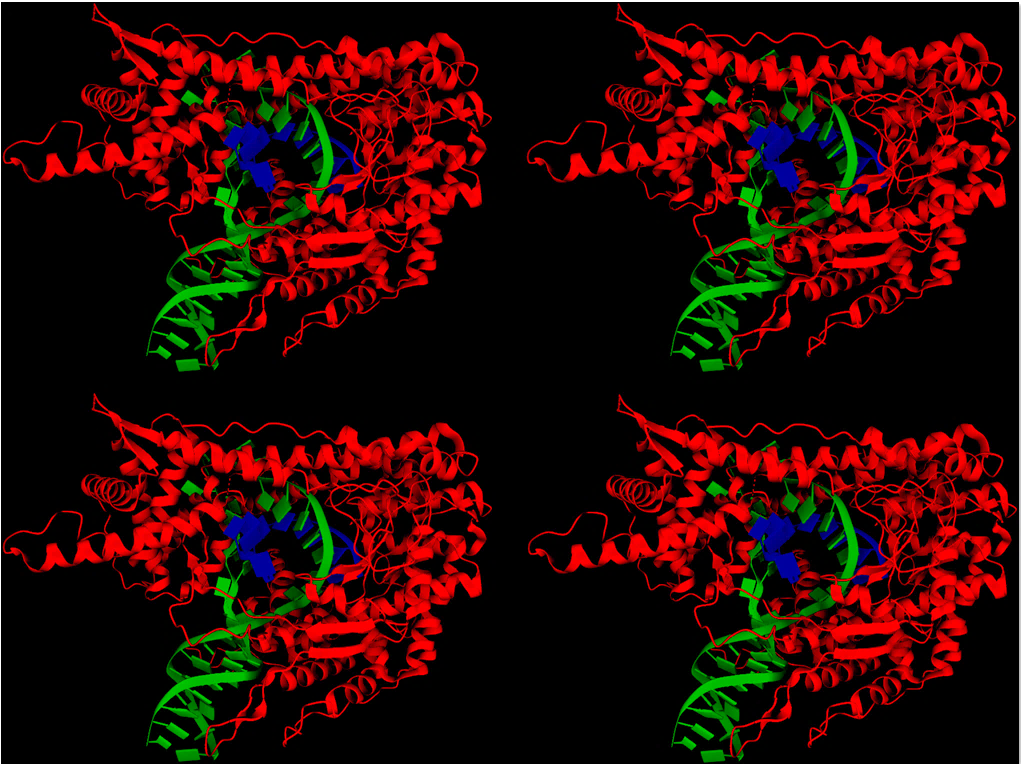 | 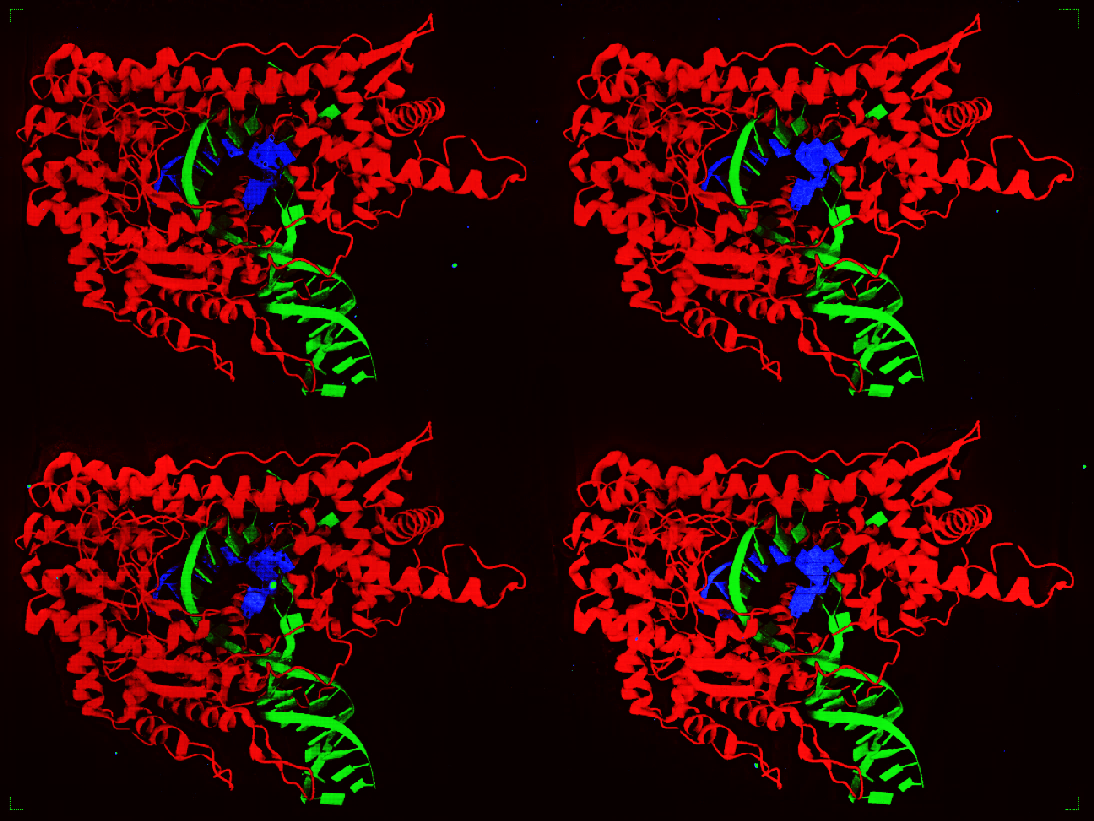 |
| --- | --- |
| **Supplementary Figure 5**. RGB image of the T7 RNA polymerase elongation complex. Original RGB image of the T7 RNA polymerase elongation complex (PDB 1MSW) as bitmap (left), and fluorescent image after synthesis of the same as a DNA template array, subsequent conversion to RNA, and hybridization to the RNA array with the complementary sequences labeled with Cy5 (red - polymerase), Cy3 (green - DNA) and fluorescein (blue - RNA); physical dimension of the RNA array is 14 × 10 mm with pixel sizes of 14 × 14 µm (right). | |

| 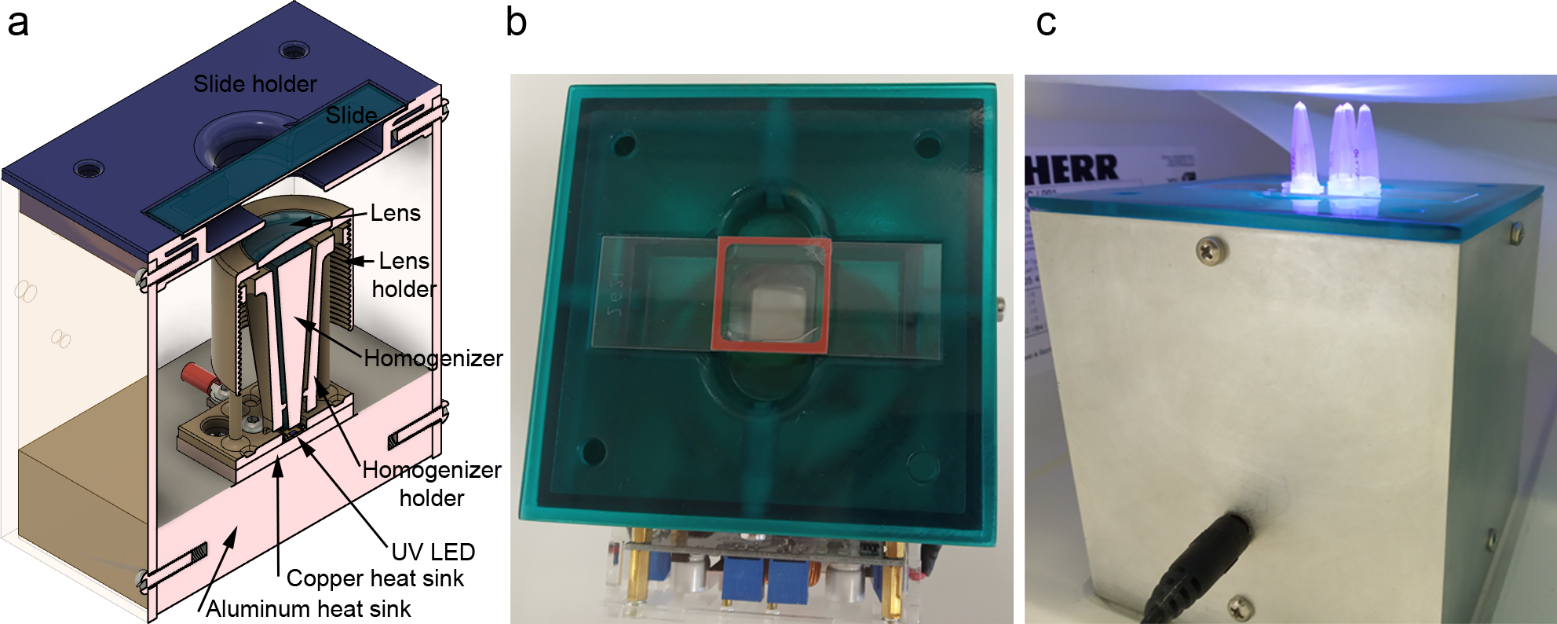 |
| --- |
| **Supplementary Figure 6**. 365 nm UV light source for microarray photocrosslinking. a) Cross-sectional view through the device. The LED and copper heat sink are described in ref. 53 (Hölz et al 2017). The light from the LED is spatially homogenized using a tapered light pipe (Edmund Optics 65-843) positioned on the LED with a 3D printed support. A 3D printed lens holder is threaded to the homogenizer holder to allow additional control of the light beam. The glass slide bearing the microarray is supported in place by a 3D printed slide holder. b) Photograph of a glass slide with a buffer-filled Grace Bio-Labs SecureSeal hybridization chamber on the UV light source. The current regulator for the LED is also visible. c) Photocrosslinking in solution using a glass slide to support UV transparent polyethylene microcentrifuge tubes with flat caps containing the experimental solution. The DC supply to the current regulator is visible. In order to facilitate photocrosslinking at 4°C, light exposure was carried out in a refrigerator after acclimatization of the box for 30 min before use. |

**Supplementary Tables**

**Supplementary Table 1**. DNA template sequences used in the high-resolution color image of T7 RNA polymerase elongation complex (Figure 1d and Supplementary Figure 5). These templates are connected to the glass surface via a common sequence, corresponding to the primer complement with a T5 linker (5′-TTCGCCGTGTCCCTATTTTT-3′), at the 3′ end.

| **Sequence (5′→3′)** | **DNA templates (-5′ truncations)** | **Color (8 bit intensity)** |
| --- | --- | --- |
| TCACCGAATCGATTCCATCTGCTTC | t3-Cy5 | Red (255) |
| ACCGAATCGATTCCATCTGCTTC | t3-Cy5-2 | Red (218) |
| GAATCGATTCCATCTGCTTC | t3-Cy5-5 | Red (182) |
| CCATCTGCTTC | t3-Cy5-14 | Red (145) |
| CATCTGCTTC | t3-Cy5-15 | Red (109) |
| ATCTGCTTC | t3-Cy5-16 | Red (72) |
| TCTGCTTC | t3-Cy5-17 | Red (36) |
| TC | t3-Cy5-23 | Red (0) |
| TCAACCCAGGTCCAATTTCC | t-Cy3 | Green (255) |
| AACCCAGGTCCAATTTCC | t-Cy3-2 | Green (218) |
| CCCAGGTCCAATTTCC | t-Cy3-4 | Green (182) |
| CCAGGTCCAATTTCC | t-Cy3-5 | Green (145) |
| CAGGTCCAATTTCC | t-Cy3-6 | Green (109) |
| AGGTCCAATTTCC | t-Cy3-7 | Green (72) |
| GTCCAATTTCC | t-Cy3-9 | Green (36) |
| CC | t-Cy3-18 | Green (0) |
| ACAGTGGATCGTACTCAGGTCTCA | t4-FAM | Blue (255) |
| ATCGTACTCAGGTCTCA | t4-FAM-7 | Blue (218) |
| GTACTCAGGTCTCA | t4-FAM-10 | Blue (182) |
| TACTCAGGTCTCA | t4-FAM-11 | Blue (145) |
| ACTCAGGTCTCA | t4-FAM-12 | Blue (109) |
| TCAGGTCTCA | t4-FAM-14 | Blue (72) |
| AGGTCTCA | t4-FAM-16 | Blue (36) |
| CA | t4-FAM-22 | Blue (0) |

**Supplementary Table 2.** Characteristics of the design for the custom 4×44K SurePrint Agilent microarray (AMADID 086693).

| Total number of features on each array: | 45220 |
| --- | --- |
| Number of Agilent controls: | 1417 |
| Percentage filled: | 84.07% 🡪 16% background features (7203) |
| Replicates per sequence: | 60 |
| Individual sequences: | 610 |
| - …with Pc + variations^a^: | 544 |
| - …with t + variations^a^: | 512 |
| - …with tc^b^: | 8 |

^a^ Variations include permutations of positions in primer complement (Pc: 5′-**TTC**GCCGTGTCCCTA-3′, permuted positions in bold) and template (t: 5′-TCAACCCAG**GTC**CAATTTCC-3′), as well as different lengths of dT linkers (T01, T05, T10, T20).

^b^ tc = template complement; sequences serving as controls, allowing for parallel hybridization to untreated DNA in one part of the array and to RNA as the product of conversion from “Pc+t” sequences in another part of the same slide

**Supplementary Table 3.** Sequences of oligonucleotide probes used in on-array experiments.

| **Name** | **Sequence (5′→3′)** | **Length** | **Chemistry** | **Modification** |
| --- | --- | --- | --- | --- |
| Primer | UAGGGACACGGCGAA | 15 nt | 2′-*O*-methyl RNA | 5′-psoralen |
| t-Cy3 | TCAACCCAGGTCCAATTTCC | 20 nt | DNA | 5′-Cy3 |
| t2-Cy3 | GATGATGTATGGCACATGATTCTATGGTAA | 30 nt | DNA | 5′-Cy3 |
| t3-Cy5 | UCACCGAAUCGAUUCCAUCUGCUUC | 25 nt | RNA | 5′-Cy5 |
| t4-FAM | ACAGTGGATCGTACTCAGGTCTCA | 24 nt | DNA | 3′-FAM |

**Supplementary Table 4**. DNA template sequences to test enzymatic primer extension in presence of Cy3-UTP. The design contains each of these sequences in two variants: either as controls, *i.e.* with the template directly 5′ to the linker, or as substrate for conversion to RNA by including a 3′-terminal sequence complementary to the primer (5′-TTCGCCGTGTCCCTA-3′). All sequences share a common T5 linker at the 3′ end.

| **Sequence (5′→3′)** | **Name** |
| --- | --- |
| TGCTGCTGCTGCTGCTGCTGCTGCTGCTGCTGCTGCTGCTGCTGCTGCTGCTGCTGCTGC**A** | A01 |
| TGCTGCTGCTGCTGCTGCTGCTGCTGCTGCTGCTGCTGCTGCTGCTGCTGCTGCTGC**A**TGC | A04 |
| TGCTGCTGCTGCTGCTGCTGCTGCTGCTGCTGCTGCTGCTGCTGCTGCTGCTGC**A**TGCTGC | A07 |
| TGCTGCTGCTGCTGCTGCTGCTGCTGCTGCTGCTGCTGCTGCTGCTGCTGC**A**TGCTGCTGC | A10 |
| TGCTGCTGCTGCTGCTGCTGCTGCTGCTGCTGCTGCTGCTGCTGCTGC**A**TGCTGCTGCTGC | A13 |
| TGCTGCTGCTGCTGCTGCTGCTGCTGCTGCTGCTGCTGCTGCTGC**A**TGCTGCTGCTGCTGC | A16 |
| TGCTGCTGCTGCTGCTGCTGCTGCTGCTGCTGCTGCTGCTGC**A**TGCTGCTGCTGCTGCTGC | A19 |
| TGCTGCTGCTGCTGCTGCTGCTGCTGCTGCTGCTGCTGC**A**TGCTGCTGCTGCTGCTGCTGC | A22 |
| TGCTGCTGCTGCTGCTGCTGCTGCTGCTGCTGCTGC**A**TGCTGCTGCTGCTGCTGCTGCTGC | A25 |
| TGCTGCTGCTGCTGCTGCTGCTGCTGCTGCTGC**A**TGCTGCTGCTGCTGCTGCTGCTGCTGC | A28 |
| TGCTGCTGCTGCTGCTGCTGCTGCTGCTGC**A**TGCTGCTGCTGCTGCTGCTGCTGCTGCTGC | A31 |
| TGCTGCTGCTGCTGCTGCTGCTGCTGC**A**TGCTGCTGCTGCTGCTGCTGCTGCTGCTGCTGC | A34 |
| TGCTGCTGCTGCTGCTGCTGCTGC**A**TGCTGCTGCTGCTGCTGCTGCTGCTGCTGCTGCTGC | A37 |
| TGCTGCTGCTGCTGCTGCTGC**A**TGCTGCTGCTGCTGCTGCTGCTGCTGCTGCTGCTGCTGC | A40 |
| TGCTGCTGCTGCTGCTGC**A**TGCTGCTGCTGCTGCTGCTGCTGCTGCTGCTGCTGCTGCTGC | A43 |
| TGCTGCTGCTGCTGC**A**TGCTGCTGCTGCTGCTGCTGCTGCTGCTGCTGCTGCTGCTGCTGC | A46 |
| TGCTGCTGCTGC**A**TGCTGCTGCTGCTGCTGCTGCTGCTGCTGCTGCTGCTGCTGCTGCTGC | A49 |
| TGCTGCTGC**A**TGCTGCTGCTGCTGCTGCTGCTGCTGCTGCTGCTGCTGCTGCTGCTGCTGC | A52 |
| TGCTGC**A**TGCTGCTGCTGCTGCTGCTGCTGCTGCTGCTGCTGCTGCTGCTGCTGCTGCTGC | A55 |
| TGC**A**TGCTGCTGCTGCTGCTGCTGCTGCTGCTGCTGCTGCTGCTGCTGCTGCTGCTGCTGC | A58 |
| **A**TGCTGCTGCTGCTGCTGCTGCTGCTGCTGCTGCTGCTGCTGCTGCTGCTGCTGCTGCTGC | A61 |

**Supplementary Table 5**. Oligonucleotide probes used to determine the efficiency of T7 RNA polymerase extension of a 2′-*O*Me-RNA primer using a ssDNA template in solution.

| **Probe Name** | **Sequence (5′→3′)** | **Length** | **Chemistry** | **Modification** |
| --- | --- | --- | --- | --- |
| Product | UAGACCAGGGUGGUUCAUGAUGAUGAC *UUACCAUAGAAUCAUGUGCCAUACAUCAUC* | 57 nt | 2′-*O*Me-RNA / *RNA* | 5′-psoralen |
| Primer | UAGACCAGGGUGGUUCAUGAUGAUGAC | 27 nt | 2′-*O*Me-RNA | 5′-psoralen |
| Template | GATGATGTATGGCACATGATTCTATGGTAA GTCATCATCATGAACCACCCTGGTCTATTTTT | 62 nt | DNA | none |
