# Supplementary material for: Simple synthesis of massively parallel RNA microarrays via enzymatic conversion from DNA microarrays
Source: Nat Commun. 2022 Jun 30;13:3772. doi: 10.1038/s41467-022-31370-9 (PMC9246885; doi:10.1038/s41467-022-31370-9)
Supplement: Supplementary file 3 — Description of Additional Supplementary Files [file 41467_2022_31370_MOESM3_ESM.docx]

**Description of Additional Supplementary Files**

**Simple synthesis of massively parallel RNA microarrays via enzymatic conversion from DNA microarrays**

File Name: Supplementary Data File

Description: Sequences of spots shown in the close-up of the 4×44K Agilent SurePrint custom DNA microarray (AMADID 086693) in Supplementary Figure 1.

File Name: Source Image Data

Description: Uncropped image of polyacrylamide gel shown in Figure 3a.
