# Supplementary material for: Simple synthesis of massively parallel RNA microarrays via enzymatic conversion from DNA microarrays
Source: Nat Commun. 2022 Jun 30;13:3772. doi: 10.1038/s41467-022-31370-9 (PMC9246885; doi:10.1038/s41467-022-31370-9)
Supplement: Supplementary file 5 — Reporting Summary [file 41467_2022_31370_MOESM5_ESM.pdf]

nature portfolio

Corresponding author(s): Mark M. Somoza

Last updated by author(s): Jun 5, 2022

Reporting Summary

Nature Portfolio wishes to improve the reproducibility of the work that we publish. This form provides structure for consistency and transparency in reporting. For further information on Nature Portfolio policies, see our Editorial Policies and the Editorial Policy Checklist.

Statistics

For all statistical analyses, confirm that the following items are present in the figure legend, table legend, main text, or Methods section.

n/a	Confirmed	
☐	☒	The exact sample size (n) for each experimental group/condition, given as a discrete number and unit of measurement
☒	☐	A statement on whether measurements were taken from distinct samples or whether the same sample was measured repeatedly
☒	☐	The statistical test(s) used AND whether they are one- or two-sided<br>*Only common tests should be described solely by name; describe more complex techniques in the Methods section.*
☒	☐	A description of all covariates tested
☒	☐	A description of any assumptions or corrections, such as tests of normality and adjustment for multiple comparisons
☒	☐	A full description of the statistical parameters including central tendency (e.g. means) or other basic estimates (e.g. regression coefficient) AND variation (e.g. standard deviation) or associated estimates of uncertainty (e.g. confidence intervals)
☒	☐	For null hypothesis testing, the test statistic (e.g. F, t, r) with confidence intervals, effect sizes, degrees of freedom and P value noted<br>*Give P values as exact values whenever suitable.*
☒	☐	For Bayesian analysis, information on the choice of priors and Markov chain Monte Carlo settings
☒	☐	For hierarchical and complex designs, identification of the appropriate level for tests and full reporting of outcomes
☒	☐	Estimates of effect sizes (e.g. Cohen's d, Pearson's r), indicating how they were calculated

Our web collection on statistics for biologists contains articles on many of the points above.

Software and code

Policy information about availability of computer code

Data collection	NimbleScan 2.1.68 and GenePix 6.1
Data analysis	Microsoft Excel 2020, OriginPro 2020 (version 9.7.0.188)

For manuscripts utilizing custom algorithms or software that are central to the research but not yet described in published literature, software must be made available to editors and reviewers. We strongly encourage code deposition in a community repository (e.g. GitHub). See the Nature Portfolio guidelines for submitting code & software for further information.

Data

Policy information about availability of data

All manuscripts must include a data availability statement. This statement should provide the following information, where applicable:

- Accession codes, unique identifiers, or web links for publicly available datasets
- A description of any restrictions on data availability
- For clinical datasets or third party data, please ensure that the statement adheres to our policy

Raw image files of microarray scans are available from the corresponding author upon a reasonable request. The design of the Agilent microarray is available through AMADID number 086693. The crystal structure of the T7 RNA polymerase elongation complex is available at http://doi.org/10.2210/pdb1MSW/pdb.

Field-specific reporting

Please select the one below that is the best fit for your research. If you are not sure, read the appropriate sections before making your selection.

- [x] Life sciences
- [ ] Behavioural & social sciences
- [ ] Ecological, evolutionary & environmental sciences

For a reference copy of the document with all sections, see nature.com/documents/nr-reporting-summary-flat.pdf

Life sciences study design

All studies must disclose on these points even when the disclosure is negative.

Sample size	No sample size calculation was performed as this was not relevant to the study.
Data exclusions	No data were excluded from the analyses, except for two data points (out of 85) in the x-axis category "7" (concerning the position of dA the template) in Fig. 3b due to a piece of dust on the surface at these positions causing extraordinarily high fluorescent signal.
Replication	The microarray experiments in Fig. 1 were performed a single time each. Both the RNase degradation assays, as shown in Fig. 2d (step 2) and Supplementary Fig. S3d, and the degradation assays using TURBO DNase (scan shown in Fig. 2d, step 1) were performed four times. PAGE analysis of primer extension in solution was carried out according to the procedure described herein independently twice to yield results as shown in Fig. 3a.
Randomization	When applicable, DNA probes for conversion to RNA were randomly distributed on the array. Otherwise no randomization was relevant because all samples were synthesized identically both into the experimental and control microarrays and then treated in parallel with either an experimental or control protocol such as DNase vs no DNase.
Blinding	Blinding was not performed and was not relevant since the outcome parameter (conversion to RNA) can be determined unambiguously.

Reporting for specific materials, systems and methods

We require information from authors about some types of materials, experimental systems and methods used in many studies. Here, indicate whether each material, system or method listed is relevant to your study. If you are not sure if a list item applies to your research, read the appropriate section before selecting a response.

Materials & experimental systems

n/a	Involved in the study	
☒	☐	Antibodies
☒	☐	Eukaryotic cell lines
☒	☐	Palaeontology and archaeology
☒	☐	Animals and other organisms
☒	☐	Human research participants
☒	☐	Clinical data
☒	☐	Dual use research of concern

Methods

n/a	Involved in the study	
☒	☐	ChIP-seq
☒	☐	Flow cytometry
☒	☐	MRI-based neuroimaging
